# Evaluating Academic Mentorship Programs in Low- and Middle-Income Country Institutions: Proposed Framework and Metrics

**DOI:** 10.4269/ajtmh.18-0561

**Published:** 2018-11-14

**Authors:** Benjamin H. Chi, Jose M. Belizan, Magaly M. Blas, Alice Chuang, Michael D. Wilson, Carla J. Chibwesha, Carey Farquhar, Craig R. Cohen, Tony Raj

**Affiliations:** 1 University of North Carolina, Chapel Hill, North Carolina;; 2 Instituto de Efectividad Clinica y Sanitaria, Buenos Aires, Argentina;; 3 Universidad Peruana Cayetano Heredia, Lima, Peru;; 4 Noguchi Memorial Institute for Medical Research, University of Ghana, Legon, Ghana;; 5 University of Washington, Seattle, Washington;; 6 University of California, San Francisco, San Francisco, California;; 7 University of California Global Health Institute, San Francisco, California;; 8 St. John’s Research Institute, Bengaluru, India

## Abstract

A growing number of low- and middle-income country (LMIC) institutions have developed and implemented formal programs to support mentorship. Although the individual-level benefits of mentorship are well established, such activities can also sustainably build institutional capacity, bridge inequities in health care, and catalyze scientific advancement. To date, however, evaluation of these programs remains limited, representing an important gap in our understanding about the impact of mentoring. Without rigorous and ongoing evaluation, there may be missed opportunities for identifying best practices, iteratively improving program activities, and demonstrating the returns on investment in mentorship. In this report, we propose a framework for evaluating mentorship programs in LMIC settings where resources may be constrained. We identify six domains: 1) mentor–mentee relationship, 2) career guidance, 3) academic productivity, 4) networking, 5) wellness, and 6) organizational capacity. Within each, we describe specific metrics and how they may be considered as part of evaluation plans. We emphasize the role of measurement and evaluation at the institutional level, so that programs may enhance their mentoring capacity and optimize the management of their resources. Although we advocate for a comprehensive approach to evaluation, we recognize that—depending on stage and relative maturity—some domains may be prioritized to address short- and medium-term program goals.

## Introduction

Significant investments have been made to foster mentorship across a range of scientific disciplines. Many examples can be found in the medical and public health literature, including university programs,^1–3^ mentored fellowships,^4,5^ and multi-institutional collaborative networks.^6–8^ Underpinning these efforts is the understanding that contextual factors—including the institutional environment—can play a determining role in mentorship success.

Despite this wealth of experience, however, there remains limited data to support specific mentoring practices. In their review of 39 studies, for example, Sambunjak et al.^9^ found that the evidence supporting the effectiveness of mentorship was not strong. They attributed this result to the poor quality of assessment data, including a predominance of cross-sectional studies, reliance on self-report surveys, lack of comparison groups, insufficient detail about the nature of mentoring relationships, and a focus on high-income country (HIC) settings. Other systematic reviews have reached similar conclusions. Across 13 mentoring programs for underrepresented minorities, Beech et al.^10^ described a relative paucity of outcome-driven evaluations. In a review of 18 studies, Kashiwagi et al.^11^ reported a larger number of evaluation metrics (e.g., mentee surveys, meeting attendance, retention rates, and academic productivity), but few long-term results.

Formal mentoring in low- and middle-income country (LMIC) institutions has grown and these programs often face opportunities and challenges very different from their HIC counterparts.^12,13^ Unfortunately, the evaluation of many programs remains limited and this represents an important gap. Evaluation activities can help establish best practices across a variety of different settings. Performed on an ongoing basis, they can also be used to systematically track progress, identify programmatic gaps, and support efforts to improve the quality of mentorship.^14^ Program evaluations can also be used to justify past investments and leverage additional resources. At this time, few—if any—consensus metrics have been recommended to assess mentorship at an institutional level, even in HIC settings such as the United States.^15^ In this report, we describe a framework for the monitoring of mentorship programs, with a particular emphasis on LMIC institutions.

## Evaluation framework

Our framework was developed in consultation with academic leaders from Africa, Asia, South America, and North America. This included two face-to-face meetings—in Middleburg, VA (2017) and in New York, NY (2018)—and several structured discussions via teleconference. Through this process, we identified six key domains relevant to mentorship evaluation: 1) mentor–mentee relationship, 2) career guidance, 3) academic productivity, 4) networking, 5) wellness, and 6) organizational capacity. Within each, we describe example metrics that may be considered in the evaluation of mentoring programs (Table 1). Because of the anticipated diversity of settings—especially in the LMIC institutions that we seek to target with this journal supplement—we do not prescribe specific program indicators. Instead, these measures are deliberately kept broad to account for the differences in local settings. Like others, we recommend a combination of objective and subjective measures.^16^ Although we describe the potential impact for individual mentor–mentee pairs, we emphasize the role of measurement and evaluation at the institutional level. This perspective has been largely overlooked in the mentorship literature; however, we believe it to be of particular importance for LMIC institutions seeking to establish or further enhance their mentoring capacity and optimize the management of their resources.

**Table 1:** Example metrics for evaluating mentorship at the individual and institutional levels, categorized according to six primary domains

Domain	Category	Individual level	Institutional level
Mentor–mentee relationship	Mentor/mentee satisfaction	Assessments should include mentor’s and mentee’s strengths and weakness, as well as their level of engagement in the relationship. Collected via surveys or interviews, such feedback can be used to improve individual mentoring relationship.	At the divisional, departmental, or school level, regular assessments can help to identify gaps in institutional support. Feedback can be used to direct specific resources toward improving engagement between mentors and mentees.
	Benefits to mentor/mentee	Articulation of the benefits of relationships—for both the mentor and mentee—can help to sustain such engagement over time.	Although mentoring inherently possesses an element of altruism, identification of the perceived benefits at the institutional level can be used to support such relationships and promote activity in this area.
	Costs to mentor/mentee	The costs associated with mentoring relationships (e.g., financial expense, time investments, and opportunity cost) should be articulated and balanced against the perceived benefits.	At an institutional level, the perceived costs of mentorship could serve as a disincentive to such activity. Barriers should be identified and actively addressed.
Career guidance	Mentee career planning	Mentors should provide guidance to mentees in identifying, mapping, and charting progress along optimal career pathways. This may include the recognition and development of specific skills. Tools such as IDPs can enhance such activity.	The assessment of mentee career planning may be limited at an institutional level. However, programs can indirectly measure this through mentee satisfaction surveys or through assessments of IDP implementation, especially around specific mentee career milestones.
	Mentee career advancement	At the individual level, successful mentorship should support career advancement, regardless of the selected track (e.g., research, education, public health, and clinical medicine). This includes appointments and promotions, as well as career productivity recognition (see *Academic productivity*).	Programs may measure their success according to articulated institutional priorities. For example, departments or schools may emphasize specific career tracks based on their institutional mission and strengths. This may also include the retention of prominent faculty and staff within the institution over time.
Academic productivity	Mentee scholarship	Productivity of mentees is commonly used as an indicator for mentorship success. When assessing an individual mentor–mentee pair, measures may include published articles, accepted conference abstracts, invited talks, funded grants, and courses taught. These metrics may also be expanded to include awards and recognition.	Although the metrics are largely the same, an aggregate assessment at the institutional level may provide the overall productivity resulting from mentoring efforts within a specific program, department, and/or school.
	Impact of mentee scholarship	Understanding the impact of mentee scholarship is critical and should be considered within the scope of academic productivity. This may be focused within the specific academic community (e.g., number of citations for an article) or encompass broader audiences (e.g., incorporation into health policy). Dissemination of work via different platforms—from traditional media to newer social media platforms—may also be considered.	Although the metrics are largely the same, an aggregate assessment at the institutional level may provide the overall productivity resulting from mentoring efforts within a specific program, department, and/or school.
Networking	Connections to new collaborators and research networks	Collaborations are an important part of professional development and mentors serve a role in facilitating such partnerships. At an individual level, however, mapping such relationships may have limited utility. Potential metrics could include collaborative scholarly activity by the mentee and leadership roles within professional societies or research networks.	At an institutional level, newer approaches such as grant mapping, coauthorship mapping, and other bibliometric analyses can demonstrate the level of engagement in the broader academic community. Many of these techniques show professional connections of the mentor and mentee, demonstrating areas of joint collaboration with others.
Wellness	Work–life prioritization	In their interactions with mentees, mentors signal expectations around work–life priorities and model specific behaviors. A general alignment of work–life priorities is needed for a successful mentoring relationship. This may be measured indirectly via satisfaction surveys (see *Mentor-mentee relationships*).	Institutions often have a prevailing culture about work–life prioritization and, where possible, this should be made explicit. Mentors should be evaluated according to these organizational expectations, as they play a critical role in institutionalizing such priorities.
	General wellness	Mentors play an important role in supporting mentee wellness. They can help trainees to identify sources of conflict between personal and professional priorities, and assist in their resolution.	At an institutional level, using validated instruments, wellness among mentors and mentees can be monitored over time. Programs should consider indirect measures as well, including job turnover over time.
Organizational capacity	Mentoring capacity	–	The pool of qualified faculty is an indicator of organizational support for mentorship. When this number is small, it may threaten the quality of mentorship.
	Organizational support	–	An inventory of institutional policies and programs surrounding mentorship can provide valuable information about organizational support. Instruments that assess the culture of mentorship at the institutional level can provide important insight.
	Diversity	–	Effective mentorship may draw on shared background and experiences. Diversity within the mentorship pool should be promoted and encouraged.
	Mentoring activities by the mentee	In academic settings, mentorship is a cornerstone of ongoing institutional success. As these individuals advance in their careers, expectations for mentorship should grow accordingly. The degree in which they engage in these activities is an indicator of organizational commitment and capacity.	Assessment of ongoing mentorship (i.e., mentees providing support to trainees of their own) should be monitored at an institutional level. Bibliometric analysis, including the mapping of mentoring relationships, can show the “downstream” impact of senior faculty (see *Networking*).

IDP = individual development plan.

### Mentor–mentee relationships.

Cultivating strong mentor–mentee relationships is critical to the success and sustainability of mentorship programs. Frameworks that have been proposed assess different aspects of the relationship; however, most share similar or overlapping areas. Pfund et al.^16^ emphasized the mentor’s role in research, interpersonal, psychosocial and career, cultural responsiveness and diversity, and sponsorship. Fleming et al. developed and validated the Mentoring Competency Assessment, a 26-item inventory that evaluates a mentor’s skills in maintaining effective communication, aligning expectations, assessing understanding, address diversity, fostering independence, and promoting professional development.^17^ Law et al.^14^ compiled other validated instruments that assess the quality of the mentoring relationship, including the Mentoring Role Instrument^18^ and Mentorship Effectiveness Scale.^19^ Such approaches are promising, especially when measured longitudinally, but will likely require adaptation for LMIC institutions. In many settings, for example, research supervision often prevails over more nuanced concepts of career and scientific mentorship. Nevertheless, newer programs that seek to change the culture of mentorship at an institutional level—including the “Mentoring the Mentors” workshops described in this supplement^20^—should consider this type of competency-based approach as an early outcome.

We also emphasize the benefits and costs related to the mentor–mentee relationship. The benefits of mentorship are well documented for mentees alone (e.g., career guidance, acquisition of skills, and expertise), mentors alone (e.g., satisfaction and reinforcement of skills), and jointly (e.g., academic outputs).^12,21^ Less explored are the costs of mentorship, especially for those serving as mentors. Typically, these are not financial in nature (although they can be), but rather of time and opportunity. Understanding the relative benefits and costs for faculty members is essential to the evaluation process. When the costs are perceived to be higher than the benefits, this could negatively influence the number of available mentors and the quality of mentorship. From an institutional perspective, changes can be made to modify this cost–benefit ratio, including recognition for outstanding mentorship, allocated effort for mentoring activities, and incorporation of mentorship into local promotion criteria.

### Career guidance.

Mentors play an important role in their mentees’ career trajectory, including guidance and support for professional development and career planning. Evaluating success for such activities can be challenging, but we envision two possible approaches. A mentor’s level of engagement can be an important metric for this domain. This may be measured subjectively via mentee satisfaction surveys (see above) or objectively via specific process indicators (i.e., mentoring outputs required by the institution or program). Instruments such as individualized development plans (IDPs) can play an important role. Most IDPs require that the mentee articulate concrete goals and expected accomplishments over a 3- to 5-year time horizon. The development and joint monitoring of an IDP facilitates discussions about career planning between mentor and mentee; it also serves as an important foundation for self-reflection. External audits of the IDP process, including the tracking of key career milestones according to planned timelines, can further enrich the evaluation of mentoring programs.

Career guidance may also be measured by the mentee’s downstream career advancement. This may include academic appointments and promotions, awards and recognition, and/or other career milestones. At an individual level, a good mentor helps the mentee identify his/her talents, supports skill development in that area, and focuses professional growth in a way that best leverages this skill set. From an institutional perspective, however, such measures of success may be interpreted more narrowly. For example, a school or department may seek to develop talented faculty along its stated priorities; the institution may further emphasize the promotion and retention of its own internal trainees and faculty.^3^ Such considerations must be clearly articulated in mentorship evaluation plans, so that expectations between mentors, mentees, and institutions are properly aligned.

### Academic productivity.

Mentorship is often judged by the yardstick of academic productivity. Metrics such as published articles, conference presentations, and funded grants are attractive because of the ease by which they can be measured. Such outputs also hold considerable weight within academic institutions and can be a deciding factor at time of promotion. Although we recognize the practical importance of such productivity metrics, we also acknowledge several limitations, especially when used as a direct indicator for mentorship. For example, the assumption underlying these measures is that a mentee’s success may be attributed to the mentoring relationship, but the extent of this may be difficult to measure. With the growing emphasis on team mentorship,^22^ the relative contributions between individual faculty—and how they receive due recognition—may further complicate the picture. Conventions for authorship order may differ by setting and across disciplines, which should be considered at the time of evaluation. Finally, academic outputs may not fully reflect the contributions of a mentor. As such, programs seeking to evaluate mentorship should not do so based solely on academic productivity; instead, this domain should be considered alongside the others proposed in our framework.

With an increasing number of outlets for disseminating information, the impact of scientific research has garnered greater attention. Newer metrics for the published literature (e.g., *h*-index and i10 index), for example, consider both the number of citations, a crude proxy for impact, and alongside the productivity of the investigator. However, even these measures of impact may be overly narrow. Incorporation of research into policies—at the local, national, or international level—is often cited as a goal of research, but such achievements may be difficult to measure in a standardized way. Although typically not peer reviewed, contributions to the “gray literature” (e.g., position papers and policy briefs) can be highly influential for decision-making. The contribution of social media to overall scientific impact also deserves consideration. By extending research to broader audiences, often outside of the traditional scientific community, such platforms provide new and novel avenues for dissemination. Whether such impact can be measured in a systematic fashion—and what this dissemination represents—requires further investigation.

### Networking.

Across expertise and geography, collaboration is increasingly important in global health. Engagement with other academicians can promote shared learning, enhance overall job satisfaction, and accelerate scientific discovery. For junior academicians, the relationship between networking and mentorship can be bidirectional. Effective mentoring is key to establishing networks that can shape one’s career development. At the same time, by increasing the pool of available faculty and peers, effective networking can enhance the quality and scope of mentorship, particularly in the setting of multidisciplinary research.^23^

At the individual level, measurement of collaborative networks is often simple—for example, describing the number of collaborators based on predefined criteria. At an organizational level, bibliometric methodologies can enhance such analyses.^24,25^ These approaches can be used to map the connections between investigators and institutions, according to different measures of academic productivity. The result can be a powerful visual representation, particularly when depicted over time. A variety of software tools are increasingly available to construct and visualize bibliometric networks. These networks may, for instance, include journals, researchers, or individual publications, and they can be constructed based on citation, bibliographic coupling, co-citation, or coauthorship relations and institution. These tools can be used to determine changes in scientific impact pre- and post-implementation of mentoring initiatives to determine trends in productivity. In general, the size of each node demonstrates the number of publications, whereas the distance between two nodes, or the width of the line between them represents the number of coauthorships or citations depending on the preset criteria.

### Wellness.

There is increasing recognition that burnout is a threat to overall productivity. Among physicians in the United States, for example, more than half report exhaustion and emotional depletion, express difficulty finding meaning in their work, and suffer from depersonalization with patients.^26^ In settings of severe resource constraint, such proportions are likely to be higher.^27,28^

The inclusion of wellness among our evaluation domains is an innovation of this mentoring evaluation framework. Mentors can provide a supportive relationship for mentees, can help them establish better work–life balance patterns, and can instruct mentees how to negotiate for a better balance in their work responsibilities to promote more meaning. With training, mentors can serve as coaches, bringing out the best in their mentees.^29^ Mentors can also actively address these in their own lives, modeling appropriate work–life management for their mentees.

Several instruments have been validated to assess aspects of wellness.^14^ These include tools to measure work-role stress, self-esteem at work, and job involvement. Questionnaires are also targeted toward occupational burnout (e.g., Maslach Burnout Inventory^30^). Some are purposefully short, designed to encourage repeated evaluation over time (e.g., Mayo Clinic Well-being Index^31^). However, at present, few are adapted to settings outside of North America and Europe.

### Organizational capacity.

The relationship between the mentor and mentee does not exist in isolation; a number of environmental factors contribute to mentoring success. Because of our focus on program evaluation at an institutional level, we highlight the important role of organizational capacity. We emphasize metrics for mentoring capacity, organizational support, and diversity. Outcomes may include the number of available mentors and the gender, ethnic, and even religious diversity of the mentor pool. Evidence that mentorship has been institutionalized—for example, mentor training programs, recognition of mentors via coauthored articles or co-funded grants, inclusion of mentorship within promotions criteria—should be regularly assessed and critically evaluated. These objective measures may be complemented by subjective data about the perceived culture of mentoring within the institution. Finally, an important indicator of organizational capacity is the self-perpetuation of mentorship. The engagement of mentees in mentoring themselves should be expected, supported, and incentivized via institutional policies. Such forward-thinking approaches can be a powerful contextual driver and demonstrate that mentorship is valued within the organization at all levels.

## Tailoring evaluations to the institutional setting

Mentorship evaluations are often considered at the individual level, where assessments can be used to strengthen relationships and take stock of mentee progress. In this commentary, however, we consider their application at the institutional level, whether it be from the perspective of the program, department, or school. In our view, there are at least three general ways in which evaluation data may be used.

First—and perhaps most practical—is the use of evaluation findings to support program planning. Properly designed, such information can highlight gaps in mentorship and provide actionable feedback for program improvement. Similar to the environmental determinants proposed by Megher et al.,^15^ such information sits at the interface between the levels of *mentor* and *institution* in the social ecological model for mentorship presented in this supplement.^32^ Methods for effectively communicating to program stakeholders, including mentees and mentors, are needed.^33^

Second, program evaluation can be used to demonstrate a return on investments into mentoring efforts. Given the multitude of factors that contribute to success, downstream metrics focusing on career advancement and academic productivity have inherent problems in terms of attribution. Although imperfect, they provide a crude measurement of program performance. Once demonstrated, such successes can increase organizational capacity to mentorship and potentially enhance the self-perpetuating cycle of mentorship within the institution.

Third, components of the program evaluation can be used to foster further scientific engagement at the institutional level. For example, the identification and mapping of mentor collaborations—as proposed in the networking domain—could itself be used to connect faculty members seeking new scientific partnerships. These domains can also be used to guide more rigorous, long-term evaluations of specific program strategies.^34^ Although attention to mentorship has grown significantly, the strength of evidence supporting specific practices remains limited.^9^

Although we advocate a broad evaluation strategy across all domains over time, the focus may also differ based on the relative maturity of the mentorship program and availability of resources. Newer initiatives, for example, may prioritize shorter term goals: high-quality mentor–mentee relationships, strong organizational capacity, and wellness among faculty and mentees. As programs become more established, this may transition to a greater emphasis on the downstream results (e.g., career advancement and academic productivity), given the time needed to measure the long-term success of program graduates. Evaluations must serve the needs of the program, which are likely to evolve as programs grow.

## Conclusion

In summary, we provide a broad framework for evaluating mentoring programs in LMICs. We describe six relevant domains—the mentor–mentee relationship, career guidance, academic productivity, networking, wellness, and organizational capacity—and potential metrics within each. We emphasize the important role each has in fostering success in academic mentorship at an institutional level and provide examples for how they may be monitored over time. In North America and Europe, there has been an important shift toward structured evaluation of mentoring programs, to strengthen the evidence in the field and demonstrate best practices. Such efforts are highly relevant to LMIC settings, especially within institutions with relatively new programs, and can be used to direct funds in the most effective and efficient manner.
